# Is new drug prescribing in primary care specialist induced?

**DOI:** 10.1186/1472-6963-9-6

**Published:** 2009-01-11

**Authors:** Stefan R Florentinus, Eibert R Heerdink, Liset van Dijk, AMG Fabiënne Griens, Peter P Groenewegen, Hubert GM Leufkens

**Affiliations:** 1 Utrecht University, Department of Pharmacoepidemiology and Pharmacotherapy, Utrecht Institute for Pharmaceutical Sciences (UIPS), Utrecht, The Netherlands; 2 NIVEL (Netherlands Institute for Health Services Research), Utrecht, The Netherlands; 3 Utrecht University, Department of Sociology and Department of Human Geography, Utrecht, The Netherlands; 4 SFK (Foundation for Pharmaceutical Statistics), The Hague, The Netherlands

## Abstract

**Background:**

Medical specialists are often seen as the first prescribers of new drugs. However, the extent to which specialists influence new drug prescribing in primary care is largely unknown.

**Methods:**

This study estimates the influence of medical specialists on new drug prescribing in primary care shortly after market introduction. The influence of medical specialists on prescribing of five new drugs was measured in a cohort of 103 GPs, working in 59 practices, over the period 1999 until 2003. The influence of medical specialists on new drug prescribing in primary care was assessed using three outcome measures. Firstly, the proportion of patients receiving their first prescription for a new or reference drug from a specialist. Secondly, the proportion of GPs prescribing new drugs before any specialist prescribes to their patients. Thirdly, we compared the time until the GP's first own prescribing between GPs who waited for prescriptions from specialists and those who did not.

**Results:**

The influence of specialists showed considerable differences among the new drugs studied. The proportion of patients receiving their first prescription from a specialist was greatest for the combination salmeterol/fluticasone (60.2%), and lowest for rofecoxib (23.0%). The proportion of GPs prescribing new drugs before waiting for prescriptions from medical specialists ranged from 21.1% in the case of esomeprazole to 32.9% for rofecoxib. Prescribing new drugs by specialists did not shorten the GP's own time to prescribing.

**Conclusion:**

This study shows that the influence of medical specialists is clearly visible for all new drugs and often greater than for the existing older drugs, but the rapid uptake of new drugs in primary care does not seem specialist induced in all cases. GPs are responsible for a substantial amount of all early prescriptions for new drugs and for a subpopulation specialist endorsement is not a requisite to initiate in new drug prescribing. This contradicts with the idea that the diffusion of newly marketed drugs always follows a two-step model, with medical specialists as the innovators and GPs as the followers.

## Background

Prescribing of newly marketed drugs is not uniformly distributed among physicians. A minority of physicians is responsible for the majority of all early prescriptions for new drugs shortly after market introduction [1-3]. The interface between primary and specialist care is an important factor in the mixture of drugs prescribed by general practitioners (GPs) [4]. In healthcare systems where GPs function as gatekeepers for accessing specialist care, like in the UK and the Netherlands, referral of patients and repeat prescribing of specialist-initiated prescription are important mechanisms through which specialists influence GP prescribing [5,6].

Two studies found GPs' prescribing behaviour to be a derivative of specialist prescribing by showing that 60–66% of all drugs prescribed by GPs were initiated by medical specialists [7,8]. However, these studies did not differentiate between new and old drugs. Tamblyn et al. found no indications that repeat prescribing of specialist prescriptions influenced the GP's prescribing of new drugs [2]. In addition, Robertson et al. noted that prescriptions for more recently introduced drugs were not more likely to have been specialist initiated than older drugs [9]. So despite the acknowledged impact that specialists have on the prescribing of existing drugs by GPs, little data are available when it comes newly marketed drugs.

Better understanding of the interplay between primary and specialist care, and thereby the mechanisms by which new drug diffuse into medical practice, contribute to the discussion on ensuring patient safety and a sustainable cost-containment in health care [10,11]. Therefore, the aim of this study was to estimate the influence of medical specialists on new drug prescribing in primary care shortly after market introduction. We were interested in three questions. Firstly, are newly marketed drugs in primary care mainly prescribed by medical specialists during the early post-marketing period? Secondly, how many GPs start prescribing new drugs before medical specialists do so? Thirdly, does specialist prescribing shorten the GP's time to adoption?

## Methods

### Study setting

In this study we used dispensing data from patients of 103 GPs who participated in the second Dutch national survey of general care (DNSGP-2), conducted by NIVEL (Netherlands Institute for Health Services Research) in 2001 [12,13]. The 103 GPs worked in 59 non-dispensing practices in all 12 provinces of the Netherlands. Dispensing data were collected by the network of the Foundation for Pharmaceutical Statistics (SFK) and covered the period 1999 until 2003 [14]. Because virtually all patients in the Netherlands designate a single pharmacy to fill prescriptions from both GPs and medical specialists, dispensing data provide an almost complete account of drug exposure in time [15].

### Study design

For this study we selected five new drugs as study cases, namely the combination of a long-acting bronchodilator and inhalation corticosteroid (ICS) salmeterol/fluticasone, the cyclo-oxygenase-2 inhibitor rofecoxib, the proton pump inhibitor esomeprazole, the long-acting anticholinergic bronchodilator tiotropium, and the lipid lowering HMG-CoA reductase inhibitor rosuvastatin. Table 1 shows relevant characteristics of these case study drugs.

**Table 1:** Characteristics of the five newly marketed drugs included in the study

New drug (*Brand name*)	Market introduction	Main indication	Reference group
Salmeterol/fluticasone (*Seretide/Advair*)	1999, May	Asthma/COPD*	Long acting β_2_-agonist and ICS
Rofecoxib (*Vioxx*)	2000, Apr	Rheumatoid arthritis	NSAIDs excl. low-dose aspirin
Esomeprazole (*Nexium*)	2000, Nov	Gastro-oesophageal reflux	Proton pomp inhibitors
Tiotropium (*Spiriva*)	2002, Jun	COPD	Ipratropiumbromide containing products
Rosuvastatin (*Crestor*)	2003, Mar	Hypercholesterolemia	HMG CoA reductase inhibitors

*COPD: Chronic Obstructive Pulmonary Disease

With the introduction of a new drug, physicians can either treat a patient with the tried and proven existing drug (if available) or the newly introduced drug. As reference drugs for the selected study case drugs we used all long-acting beta-2 antagonists and ICS for the combination salmeterol/fluticasone, all ipratropium bromide containing products for tiotropium, all non-steroidal anti-inflammatory drugs (NSAIDs) (excl. low-dose aspirin) for rofecoxib, all proton pump inhibitors for esomeprazole and all HMG-CoA reductase inhibitors for rosuvastatin. All five new drugs showed rapid market introduction and were listed within one year after market introduction in the top 10 drugs associated with the fastest growing expenditures in the Netherlands [14]. All drugs were fully reimbursed by all health insurance companies and could be prescribed without limitation by both GPs as medical specialists.

For this study we included patients starting on a new or a reference drug during the first six months after market introduction. We included both new starters and switchers. The date of the patient's first prescription for either the new or reference drug was termed the index date. Starting was defined as receiving a prescription for a new or reference drug and no prescription for that same drug during the six months before the index date. Patients in whom dispensing follow-up of less than six months was present were excluded. Market introduction was defined as the date of the first prescription for the new drug in the database.

### Main outcome measurements

We recorded the type of physician (GP or medical specialist) using the patient's prescription for a new or reference drug on the index date. To answer the first research question, we calculated per new drug the proportion of patients receiving their first prescription for a new drug from a medical specialist out of the total number of patients receiving the drug from both GPs and medical specialists. The same proportion was calculated for the reference groups to calculate a relative rate. To answer the second research question of how many GPs start prescribing new drugs before waiting for any prescriptions of medical specialists, we calculated the proportion of GPs that started prescribing the new drug before any of their patients received the drug from a medical specialist. To answer the third research question we calculated for each GP the time between market introduction and the date on which the GP prescribed the new drug for the fist time to a patient who never used the drug before. We compared the time to prescribing between GPs that initiate therapy before one of their patients received the drug from a medical specialist and GPs that waited for specialist to prescribe first before prescribing themselves.

## Results

In total 1,687 patients received one of the five new drugs during the first six months after market introduction. Most patients received rofecoxib (N = 596), followed by tiotropium (N = 556), rosuvastatin (N = 212), the combination salmeterol/fluticasone (N = 171) and esomeprazole (N = 152). The average age was 61.7 years (SD 15.3 years) and 57.2% was female. Overall 16,797 patients received a drug from the reference groups (mean age 51.9 years (SD 18.7); 58.8% female). Complete dispensing data were available for 80 GPs for the combination salmeterol/fluticasone. The number of included GPs for rofecoxib was 85, 90 for esomeprazole, 98 for tiotropium, and 94 for rosuvastatin.

During the first six months following market launch, three drugs were more frequently prescribed by GPs than by medical specialists. Most patients starting on rofecoxib, esomeprazole, or rosuvastatin, received their first prescription from their own GP. The proportion of patients receiving their first prescription from a GP was 77.0% for rofecoxib, 65.1% for esomeprazole, and 58.0% for rosuvastatin. On the other hand, tiotropium and salmeterol/fluticasone were more frequently initiated by medical specialists. Of the patients starting on tiotropium, 52.7% received their first prescription from a specialist. The proportion was 60.2% for salmeterol/fluticasone. Table 2 shows for each new drug the number of patients receiving a new or reference drug and the corresponding likelihood of receiving a new drug from a specialist compared to a reference drug. Except for rosuvastatin, receiving a new drug from a specialist was more likely than receiving a reference drug from a GP. The relative rate was greatest for tiotropium (RR 3.64; 95% CI 3.03–4.36) and salmeterol/fluticasone (RR 3.56; 95% CI 3.03–4.17). For rosuvastatin, no difference was observed (RR 1.06 95% CI 0.89–1.27).

**Table 2:** New drug prescribing on patient level.

	New drug N (%)	Reference drug N (%)	Relative Rate (95% CI)
	
**Salmeterol/fluticasone**			
Medical specialist	103 (60.2%)	302 (16.9%)	3.56 (3.03–4.17)
GP	68 (39.8%)	1,481 (83.1%)	Ref.
**Rofecoxib**			
Medical specialist	137 (23.0%)	1,445 (13.0%)	1.77 (1.51–2.06)
GP	459 (77.0%)	9,656 (87.0%)	Ref.
**Esomeprazole**			
Medical specialist	53 (34.9%)	424 (19.6%)	1.78 (1.41–2.25)
GP	99 (65.1%)	1,741 (80.4%)	Ref.
**Tiotropium**			
Medical specialist	293 (52.7%)	123 (14.5%)	3.64 (3.03–4.36)
GP	263 (47.3%)	726 (85.5%)	Ref.
**Rosuvastatin**			
Medical specialist	89 (42.0%)	355 (39.5%)	1.06 (0.89–1.27)
GP	123 (58.0%)	544 (60.5%)	Ref.
**Overall**			
Medical specialist	675 (40.0%)	2,649 (15.8%)	2.53 (2.37–2.71)
GP	1,012 (60.0%)	14,148 (84.2%)	Ref.

Number of patients receiving their first prescription for a new or a reference drug from a medical specialist or GP six months after market introduction.

The proportion of GPs with at least one patient in their practice that received a new drug during the first six months after market introduction ranged from 53.3% for esomeprazole to 94.9% for tiotropium (Table 3). Not all GPs initiated in prescribing the new drugs themselves. The proportion of GPs starting therapy ranged from 30.0% for esomeprazole to 66.3% for tiotropium. A substantial proportion of GPs that started prescribing new drugs did so before any of their own patients received a prescription from a specialist. The proportion of GPs prescribing new drugs before a specialist prescription ranged from 21.1% for esomeprazole to 32.9% for rofecoxib.

**Table 3:** New drug prescribing on GP level.

	Total number of GP	GPs with at least one patient in their practice receiving new drug	GPs initiating therapy with new drugs	GPs initiating therapy without waiting for a specialist prescription
Salmeterol/fluticasone	80	59 (73.8%)	34 (42.5%)	23 (28.8%)
Rofecoxib	85	68 (80.0%)	55 (64.7%)	28 (32.9%)
Esomeprazole	90	48 (53.3%)	27 (30.0%)	19 (21.1%)
Tiotropium	98	93 (94.9%)	65 (66.3%)	26 (26.5%)
Rosuvastatin	94	61 (64.9%)	34 (36.2%)	23 (24.5%)

Number GPs with at least one patient in their practice receiving a new drug, GPs initiating therapy with a new drug, and GPs initiating new drug therapy without waiting for a specialist prescription.

The time between market introduction and actual prescribing of the different new drugs showed considerable variation among GPs (Table 4). For all new drugs, the average time to prescribing was shorter (not significantly) for GPs that started prescribing before one of their patients received a prescription from a specialist compared to GPs waiting for a prescription from a medical specialist.

**Table 4:** The influence of medical specialist prescribing on the average number of days (SD) to the GP's first prescription for a new drug in the first six months after market introduction.

	GPs starting therapy before repeating a specialist prescription		GPs starting therapy after repeating a specialist prescription		
	
	N	Mean days (SD)	N	Mean days (SD)	Pearson Chi-square
Salmeterol/fluticasone	23 (67.6%)	88.6 (36.3)	11 (32.4%)	109.0 (41.3)	0.69
Rofecoxib	28 (50.9%)	71.7 (56.1)	27 (49.1%)	87.8 (42.5)	0.56
Esomeprazole	19 (70.4%)	53.8 (43.8)	8 (29.6%)	87.3 (48.9)	0.56
Tiotropium	26 (40.0%)	45.0 (46.5)	39 (60.0%)	88.0 (52.7)	0.60
Rosuvastatin	23 (67.6%)	43.1 (40.1)	11 (32.4%)	108.8 (48.1)	0.34

## Discussion

The primary objective of this study was to estimate the influence of medical specialists on new drug prescribing in primary care shortly after market introduction. This study shows that the influence of medical specialists is clearly visible for all new drugs and often greater than for the existing older drugs, but the rapid uptake of new drugs in primary care does not seem specialist induced by definition and very much drug dependent. A substantial proportion of GPs that prescribe new drugs do so without awaiting specialist prescribing.

The main advantage of this study was the possibility to identify within individual GP practices patients that received a new drug from a specialist and those receiving a prescription from their own GP. This clear distinction enabled us to estimate the influence of medical specialists on new drug prescribing in primary care. In addition, we measured the specialists' influence on new drug prescribing shortly after a new drug's market introduction in comparison to their influence on the prescribing of drugs from the same therapeutic category already present on the market.

Research on diffusion of innovations postulated a two-step model by which innovations are adopted by a population with innovators as the individuals who adopt first, followed by others copying their behaviour [16,17]. Because GPs regard medical specialists as the opinion leaders in specific medical area, they often mention specialists as the early prescribers of new drugs [18,19]. It is therefore reasonable to assume that the diffusion of new drugs also follows this two-steps model with medical specialists as innovators and GPs as followers. However, our data show that this is not the case for all new drugs. Even in the early post-marketing period in which new drug prescribing by specialists should be predominant, GP prescribing outweighed specialist prescribing in three out of the five new drugs. Moreover, we noted that specialist endorsement was not a requisite for a subpopulation of GPs. Especially rofecoxib, and in a lesser extent rosuvastatin and esomeprazole, was adopted by the majority of GPs and 32.9% adopted before ever having seen a specialist prescription for rofecoxib. Although the influence of specialists is clearly visible, GPs are innovators too.

The level of specialist prescribing differs per new drug. Especially for rosuvastatin, esomeprazole, and rofecoxib, GP were in most cases the first prescribers. On the other hand, tiotropium and the combination salmeterol/fluticasone were mostly initiated by specialists. This is in line with other studies [8,9,20,21]. Robertson et al. noted in a study among 88 Australian GPs that the proportion of specialist-initiated prescriptions ranged from 8% to 85% for different drug classes [9]. The smaller influence of medical specialists for rosuvastatin, esomeprazole, and rofecoxib may partly be explained by extensive marketing and the relative low perceived risk associated with prescribing of these drugs [22-24]. Extensive marketing campaigns may have resulted in less reluctance of GPs to adopt the new drugs fast. Moreover, rofecoxib, esomeprazole, and rosuvastatin are clear examples of new drugs showing rapid uptake in primary care that is not specialist induced.

We found that for four new drugs the likelihood of receiving a new drug from a medical specialist was significantly higher than receiving a reference drug. Only for rosuvastatin, no difference was observed. The difference between drugs may partly be explained by differences between patients receiving new drugs and those receiving reference drugs. In general, medical specialists treat a different patient mix primarily composed of more severely ill patients that may be more likely to benefit from new drugs [25,26]. In previous studies on new drug prescribing we have identified channelling of new marketed drugs in high-risk patients and those with poor a response existing therapies [25-27].

Based on the classical model of innovation, specialist prescribing could serve as a catalyst for GPs to try out the new drugs themselves. Rapid prescribing of newly marketed drugs by specialists could convince GPs through a learning-by-demonstration effect to adopt too. However, this is not confirmed by our data showing that the time to first prescription was not shorter for GPs who awaited specialist prescribing compared those prescribing before any of their patients received the drug from a medical specialist. The absence of any differences may partly be explained by seemingly great willingness among GPs to prescribe these newly marketed drugs.

The findings in this study need to be interpreted in light of its limitations. Firstly, the results are based on five new drugs and this should be taken into account when generalising the results to all new drugs. Our dispensing data contained no information on the diagnosis that may have influenced the likelihood the patient may have referred to medicals. Furthermore, we used dispensing data as a proxy of physician prescribing. Patients do not fill all prescriptions they receive from their physician in a pharmacy. It is therefore possible that the dispensing data are conservative estimations of the real prescribing of physicians. However, we had no indications that the filling rate of patients was more selective for one of the five new drugs or differed between GPs and specialists. Hospital pharmacies that dispense medication to patients visiting the hospital is still limited in the Netherlands. It is therefore unlikely that we missed prescriptions of specialists to the patients of the study GPs.

## Conclusion

This study shows that the influence of medical specialists is clearly visible for all new drugs and often greater than for older drugs, but the rapid uptake of new drugs in primary care does not seem specialist induced in all cases. GPs are responsible for a substantial amount of all early prescriptions for new drugs and for a subpopulation specialist endorsement is not a requisite to initiate in new drug prescribing. This contradicts with the idea that the diffusion of newly marketed drugs always follows a two-step model, with medical specialists as the innovators and GPs as the followers.

## Competing interests

The authors declare that they have no competing interests.

## Authors' contributions

SRF carried out the data analyses, preformed the statistical analyses, contributed to the data collection, drafted the manuscript, and participated in the study design. ERH contributed to the data collection, participated in the study design, carried out the data analyses. LvD contributed to the data collection, preformed the statistical analyses, and participated in the study design. AFG contributed to the data collection and contributed to the interpretation of the data. PPG contributed to the data collection, participated in the study design, conceived of the study, and coordinated the study. HGL contributed to the data collection, participated in the study design, conceived of the study, and coordinated the study. All authors read and approved the final manuscript.

## Pre-publication history

The pre-publication history for this paper can be accessed here:



## References

[B1] Inman W, Pearce G (1993). Prescriber profile and post-marketing surveillance. Lancet.

[B2] Tamblyn R, McLeod P, Hanley JA, Girard N, Hurley J (2003). Physician and practice characteristics associated with the early utilization of new prescription drugs. Med Care.

[B3] Leufkens HG, Urquhart J (1993). Prescriber profile and postmarketing surveillance. Lancet.

[B4] Pryce AJ, Heatlie HF, Chapman SR (1996). Buccaling under the pressure: influence of secondary care establishments on the prescribing of glyceryl trinitrate buccal tablets in primary care. BMJ.

[B5] Avery AJ, Rodgers S, Heron T, Crombie R, Whynes D, Pringle M, Baines D, Petchey R (2000). A prescription for improvement? An observational study to identify how general practices vary in their growth in prescribing costs. BMJ.

[B6] Cochrane RA, Mandal AR, Ledger-Scott M, Walker R (1992). Changes in drug treatment after discharge from hospital in geriatric patients. BMJ.

[B7] de Vries CS, van Diepen NM, Tromp TF, de Jong-van den Berg LT (1996). Auditing GPs' prescribing habits: cardiovascular prescribing frequently continues medication initiated by specialists. Eur J Clin Pharmacol.

[B8] Feely J, Chan R, McManus J, O'Shea B (1999). The influence of hospital-based prescribers on prescribing in general practice. Pharmacoeconomics.

[B9] Robertson J, Fryer JL, O'Connell DL, Sprogis A, Henry DA (2001). The impact of specialists on prescribing by general practitioners. Med J Aust.

[B10] Friedman MA, Woodcock J, Lumpkin MM, Shuren JE, Hass AE, Thompson LJ (1999). The Safety of Newly Approved Medicines: Do Recent Market Removals Mean There Is a Problem?. JAMA.

[B11] Wood AJJ (1999). The Safety of New Medicines: The Importance of Asking the Right Questions. JAMA.

[B12] Westert GP, Schellevis FG, de Bakker DH, Groenewegen PP, Bensing JM, Zee J van der (2005). Monitoring health inequalities through general practice: the Second Dutch National Survey of General Practice. Eur J Public Health.

[B13] Cardol M, Groenewegen PP, de Bakker DH, Spreeuwenberg P, van Dijk L, Bosch W van den (2005). Shared help seeking behaviour within families: a retrospective cohort study. BMJ.

[B14] Tinke J, Griens A (2001). Facts and Figures 2001.

[B15] Herings RM, Bakker A, Stricker BH, Nap G (1992). Pharmaco-morbidity linkage: a feasibility study comparing morbidity in two pharmacy based exposure cohorts. J Epidemiol Community Health.

[B16] Coleman JS, Katz E, Menzel H (1966). Medical Innovation: A Diffusion Study.

[B17] Rogers E (1995). Diffusion of Innovations.

[B18] Jacoby A, Smith M, Eccles M (2003). A qualitative study to explore influences on general practitioners' decisions to prescribe new drugs. Br J Gen Pract.

[B19] Jones MI, Greenfield M, Bradley CP (2001). Prescribing new drugs: qualitative study of influences on consultants and general practitioners. BMJ.

[B20] Himmel W, Tabache M, Kochen MM (1996). What happens to long-term medication when general practice patients are referred to hospital?. Eur J Clin Pharmacol.

[B21] Robertson J, Treloar CJ, Sprogis A, Henry DA (2003). The influence of specialists on prescribing by GPs. A qualitative study. Aust Fam Physician.

[B22] Burton B (2004). AstraZeneca digs in over advertising criticisms. BMJ.

[B23] Florentinus SR, Heerdink ER, Klungel OH, de Boer A (2004). Should rosuvastatin be withdrawn from the market?. Lancet.

[B24] Josefson D (2001). FDA warns Merck over its promotion of rofecoxib. BMJ.

[B25] Florentinus SR, Nielsen MW, van Dijk L, Leufkens HG, Hansen EH, Heerdink ER (2005). Patient characteristics associated with prescribing of a newly introduced drug: the case of rofecoxib. Eur J Clin Pharmacol.

[B26] Florentinus SR, Heerdink ER, de Boer A, van Dijk L, Leufkens HG (2005). The trade-off between cardiovascular and gastrointestinal effects of rofecoxib. Pharmacoepidemiol Drug Saf.

[B27] Leufkens HG, Urquhart J, Stricker BH, Bakker A, Petri H (1992). Channelling of controlled release formulation of ketoprofen (Oscorel) in patients with history of gastrointestinal problems. J Epidemiol Community Health.

